# Chemical Patterning on Nanocarbons: Functionality Typewriting

**DOI:** 10.3390/molecules28248104

**Published:** 2023-12-15

**Authors:** Zhongjie Huang

**Affiliations:** State Key Laboratory for Modification of Chemical Fibers and Polymer Materials, College of Materials Science and Engineering, Donghua University, Shanghai 201620, China; huangzhongjie@dhu.edu.cn

**Keywords:** molecular patterning, nanocarbon, carbon nanotube, graphene, surface functionalization, lithography

## Abstract

Nanocarbon materials have become extraordinarily compelling for their significant potential in the cutting-edge science and technology. These materials exhibit exceptional physicochemical properties due to their distinctive low-dimensional structures and tailored surface characteristics. An attractive direction at the forefront of this field involves the spatially resolved chemical functionalization of a diverse range of nanocarbons, encompassing carbon nanotubes, graphene, and a myriad of derivative structures. In tandem with the technological leaps in lithography, these endeavors have fostered the creation of a novel class of nanocarbon materials with finely tunable physical and chemical attributes, and programmable multi-functionalities, paving the way for new applications in fields such as nanoelectronics, sensing, photonics, and quantum technologies. Our review examines the swift and dynamic advancements in nanocarbon chemical patterning. Key breakthroughs and future opportunities are highlighted. This review not only provides an in-depth understanding of this fast-paced field but also helps to catalyze the rational design of advanced next-generation nanocarbon-based materials and devices.

## 1. Introduction

Carbon, the backbone of all known life on Earth, plays a pivotal role in a multitude of natural and synthetic processes. Historically, the discovery and creation of new generations of carbon allotropes and hydrocarbons have always paved the way for new science and technology [1,2,3,4,5]. In recent decades, low-dimensional nanocarbon materials have garnered significant research interests. These nanomaterials are defined by their remarkable physicochemical attributes, which are intrinsically linked to their unique low-dimensional structures. Notably, the group of materials comprise zero-dimensional fullerenes, one-dimensional (1D) carbon nanotubes (CNTs), and two-dimensional (2D) graphene [3,6,7,8], each distinguished by sp^2^-hybridized carbon atom networks conferring extraordinary electronic [9,10,11,12], optical [13,14,15], mechanical [16,17], and thermal properties [18]. Graphene, for example, exhibits unparalleled thermal conductivity and mechanical strength. In another example, single-walled carbon nanotubes (SWCNTs) demonstrate carrier mobility that markedly eclipses that of conventional silicon, alongside notable mechanical flexibility, and resilience [11,19]. Furthermore, the non-conjugated counterparts, such as nanodiamonds [20], carbon dots [21,22], and graphene oxides [23], possess their own set of unique, distinctive properties. Consequently, nanocarbons show tremendous potential in a broad spectrum of cutting-edge scientific and technological fields, including but not limited to, nanotechnology, nanoelectronics, optics, biomedical, energy, and quantum technologies [19,24].

Characterized by unprecedented surface areas, nanocarbons such as graphene and SWCNTs are quintessential quantum materials, whose surface properties are fundamental to the overall materials’ performance, with even minuscule modifications holding the transformative potential for alternative states and forms. Herein lies the promise of chemical functionalization—a tailored approach to precisely alter the low-dimensional materials’ properties, enabling controlled manipulation of energy band diagrams, synthesis of novel derivatives, and the modification of materials’ surface and interfacial properties [3,7,19,25,26,27,28]. The exploration into the chemical functionalization of nanocarbons, especially on graphene and CNTs, has been extensive and leveraged in a myriad of applications such as processing [29], coating [30], and composites [31,32,33,34]. An area of burgeoning interest is site-selective functionalization—its implications for patterning are significant, suggesting future directions for on-chip fabrication and scalable integration. The molecular functionalities and engineered defects arising from such spatial resolvable modification strategies play an indispensable role in dictating local properties of low-dimensional materials, cementing their status as a burgeoning frontier in materials science research.

The reactions employed in the functionalization of different nanocarbon surfaces often share many similarities, because they are based on a similar sp^2^-conjugated carbon structure. Combining these in one comprehensive review is valuable for the whole nanocarbon community. Despite some focus on precise modifications of nanocarbons like graphene, a clear comparison and detailed review that brings together findings from different nanocarbon fields, looking at shared challenges and subtle differences, is clearly missing and greatly needed. This review endeavors to elucidate the latest cutting-edge advances in the realm of molecular and functional patterning upon a variety of nanocarbon substrates. Section 2 underscores the developments in the chemical patterning strategies on CNTs, an area that has traditionally been underrepresented in previous reviews. Section 3 is devoted to delineating the recent advancements in molecular patterning techniques applied to graphene. Section 4 scrutinizes the novel breakthroughs achieved on alternative nanocarbon segments. Then, a perspective is given to outline the ongoing and future research opportunities and directions in Section 5. Finally, the paper concludes with a summary of observations and insights. Please note that this review is concerted on the arena of molecular patterning on nanocarbon materials, whereas patterning of these nanocarbons themselves onto other substrates has been expansively investigated elsewhere, for which the readers are referred to the extant review literature on this subject [35,36].

## 2. Chemical Patterning on CNTs

CNTs, synthetic allotropes of carbon with sp^2^ hybridization, exhibit a hexagonal lattice reminiscent of graphene but manifest in a tubular configuration. The intrinsic electrical properties of SWCNTs are governed by their structural attributes, including chirality—a defining characteristic detailing the angular alignment of the graphene lattice—which imbues SWCNTs with metallic or semiconducting behavior. Over the past three decades, surface functionalization of CNTs has undergone extensive investigation, embracing both covalent and non-covalent strategies [28,37,38,39,40,41,42], enriching the versatility of these materials across an array of applications such as compositing [43], sensing devices, and materials processing. Nevertheless, the ramifications of functionalization on the fundamental electronic and optical properties of CNTs remained obscure until recent breakthroughs.

**Figure 1:**
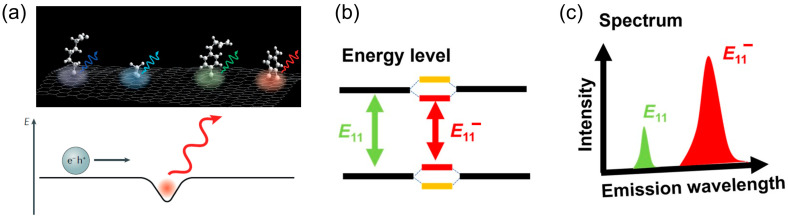
sp^3^ defects serving as OCCs on s-SWCNTs. (**a**) Engineered defect site constitutes a quantum well capable of trapping excitons and emitting bright photons. Reproduced with permission [45]. Copyright 2019, Springer Nature. The effect of the defect on the (**b**) energy diagram and (**c**) the PL spectrum. A red shifted peak in the NIR region is associated with OCC emission.

Significant progress has been made recently. It has been found that precise control of the chemical functionalization, by introducing O dopants into the sp^2^ lattice of tube walls or intentionally synthesizing sp^3^ defects (see Figure 1a) [44,45], enables the fine-tuning of the local energy level within the semiconducting SWCNTs (s-SWCNTs). S-SWCNTs, being a direct bandgap semiconductor with luminescent properties and a characteristic 1D quantum system, transform defects—from traditional nuisances in crystalline substrates to pivotal molecular focal point for regulating electrons, excitons [13], phonons, and spins. As elucidated in Figure 1, the engineered defect site constitutes a quantum well capable of trapping excitons, emitting bright photons in the near-infrared (NIR) region and serves as a room-temperature single-photon source. Consequently, such defect sites have been conferred the designation of fluorescent quantum defects or organic color centers (OCCs). The spectral emission of certain OCCs, as illustrated in Figure 1b,c, is affected by both the depth of the potential well and the original bandgap of the s-SWCNT, and thus is completely tunable by the combination of grafted moiety and the chirality of the s-SWCNT [46].

**Figure 2:**
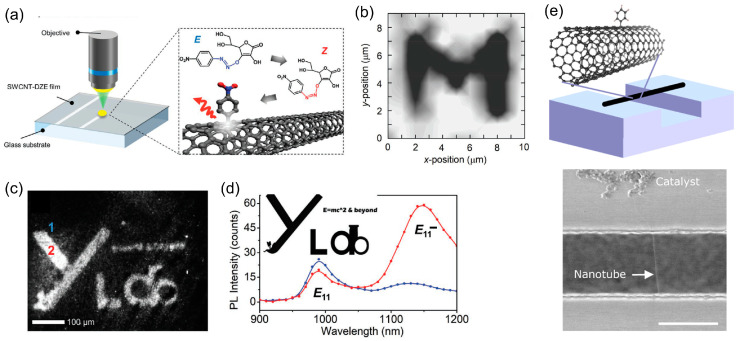
Patterning OCCs on s-SWCNTs. (**a**–**d**) Through a light-driven diazoether chemistry. Reproduced with permission [48]. Copyright 2020, Wiley-VCH. (**a**) Photolithographic patterning of OCCs on SWCNTs induced by p-nitrobenzenediazoascorbic acid and localized light exposure on SWCNTs, resulting in the formation of nitroaryl OCCs. (**b**) Spatial mapping of the D/G intensity ratio on a SWCNT film with p-nitroaryl OCCs patterned to form the letter “M”. Darker areas correspond to elevated D/G ratios. (**c**) OCC PL image of a film with a custom logo pattern, taken through a 1100 nm long-pass filter. (**d**) Corresponding site-specific PL spectra from the designated regions “1” (blue) and “2” (red) in (**c**). (**e**) Vapor-phase functionalization of air-suspended SWCNT by a UV-activated iodobenzene chemistry. Illustrated is a SWCNT bridging a trench in a silicon substrate, with post-functionalization scanning electron micrograph highlighting catalyst particles atop and the SWCNT location marked by an arrow. Reproduced with permission [52]. Copyright 2022, Springer Nature.

The localized nature of OCCs builds up strong momentum for site-selective functionalization for on-chip application. An innovative strategy to pattern OCCs on the single-chirality s-SWCNT film with a light-driven direct-writing approach was reported, as depicted in Figure 2a. This protocol harnessed light to drive the localized interaction between the *E*-isomer of p-nitrobenzenediazoascorbic acid (DZE) and SWCNTs. Remarkably, DZE exhibits chemical inertness in the absence of light at ambient conditions given the *E*-isomer’s thermodynamic stability, yet upon illumination of the SWCNTs, becomes prompted to transition into the highly reactive *Z*-isomer [47]. Leveraging this proprietary diazoether chemistry, pre-programmed patterns of aryl functional groups can thus be directly inscribed onto the SWCNT thin film under the guidance of light [48]. The spatial resolution of this chemical patterning is precise, achieving the feature size at a 1 μm scale, as evidenced by the Raman D/G ratio contour mapping (Figure 2b)—a metric indicative of the sp^3^ functionalization degree on CNTs [49,50,51]. By employing this technique, custom NIR emission patterns were successfully synthesized and recognized through NIR photoluminescence (PL) imaging (Figure 2c). A comparative PL spectral analysis between non-irradiated (denoted as “1” in Figure 2c) and irradiated (denoted as “2”) regions of a (6,5)-SWCNT film was presented in Figure 2d. The genesis of OCCs was unambiguously demonstrated by the emergent *E*_11_^−^ emission feature around ~1150 nm, with its peak intensity exhibiting a positive correlation with the duration of irradiation. This work paves the way for manipulating energy band diagram of nanocarbon, and patterning of emitters and other functional entities at the molecular scale.

As shown in the case of s-SWCNTs, the grafted molecules generate localized quantum wells which turn out to be single-photon emitting centers. In fact, single-photon sources are the key building blocks for solid-state quantum technologies, and OCCs manifest several distinctive advantages, including (i) operability under ambient temperature, (ii) high purity of single-photon emission, (iii) tunable emission wavelengths within the telecommunication band, and (iv) rich molecular and chemical tunability. These concurrent characteristics remain unparalleled in alternative material systems. Therefore, the successful patterning of OCCs demonstrates an important step towards the on-chip fabrication of quantum circuits. Furthermore, the synthesis of OCCs can be advanced through a light-driven vapor-phase reaction employing iodobenzene (Figure 2e) [52], extending the potential for more streamlined quantum circuit construction.

CNTs are blessed with versatile electronic properties. Besides the well-sorted single-chirality film as shown in Figure 2, films composed of CNTs with mixed electronic structures are prevalent in film devices. Consequently, there exists a pronounced necessity for a facile and scalable approach to incorporate CNTs within electronic devices. Wang et al. advanced a distinctive CNT lithographic technique capable of patterning both semiconducting channels and conductive pathways upon an insulating nanotube thin film, exploiting reversible functionalization of the outer walls of double-walled carbon nanotubes (DWCNTs) [53]. Pristine DWCNTs are conductive; conversely, after the functionalization of the outer walls, the film becomes insulating. Thus, by the selective erasing of surface functional groups with light resonating with the inner tube, one can generate conductive or semiconducting pathways. Experiments on power dependence exposed a critical power threshold, below which no defunctionalization occurred. An array of dots and conductive pathways were successfully patterned, and this selective molecular annihilation was attributed to the local heating of the inner tube [53].

**Figure 3:**
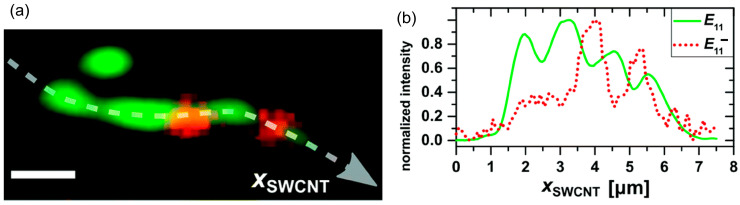
PL images of individual functionalized SWCNT. (**a**) Image of the pristine emission (green) and defect emission (red). Scale bar equals 1 μm. Corresponding intensity profiles captured along the SWCNT axis (*X*_SWCNT_) delineated by white dashed lines in (**a**) and presented in (**b**), revealing an inverse relationship between green and red signal. Reproduced with permission [54]. Copyright 2015, The Royal Society of Chemistry.

Besides molecular “writing”, the ability to “read” molecules at fine resolution provides a wealth of new insights to the nanocarbon-related chemistry and physics. Exciton trapping at OCCs, as displayed in Figure 3, can be directly visualized as hot spots in PL mapping images. While the PL associated with the *E*_11_ transition emanates nearly uninterruptedly across the whole nanotube, the one correlated to the *E*_11_^−^ is localized within the diffraction limit. Notably, the spectral emissions corresponding to the *E*_11_ and *E*_11_^−^ presented in green and red, respectively, show a complementary spatial distribution along the nanotube [54], despite the shared exciton source, which provides strong evidence to the exciton diffusion and defect-trapping model associated with OCC.

## 3. Chemical Patterning on Graphene

Graphene, a single monolayer lattice of sp²-hybridized carbon atoms, has seized huge research attention due to its distinctive electronic properties. This two-dimensional material inaugurates an entirely novel class of materials defined by atomic-scale thinness, providing groundbreaking explorations in low-dimensional physics—a field that persistently unveils startling phenomena and proliferates applications [2,3,55,56]. In addition, few-layer structured graphene has demonstrated a potential for a further tuning of electronic states through interlayer interactions.

The chemical functionalization of graphene has been extensively explored. The covalent modification of graphene surfaces has been identified as a viable route to modulate its electronic structure (e.g., opening up the bandgap [7]), enhancing its chemical reactivity, and processability, critical for the integration of graphene into complex systems. The molecular patterning on the graphene surface has also achieved significant progress. This paper only focuses on the latest breakthrough (in the past three years) or the work that has rarely been reviewed. The readers are referred to existing reviews for a better understanding of the history of chemical patterning on graphene surfaces [8,57,58,59,60,61]. The intrinsic chemical inertness attributed to graphene’s π-conjugation imposes a reticence toward chemical modification of its basal plane; hence, advanced chemistry and strategies are needed to overcome this obstacle.

**Figure 4:**
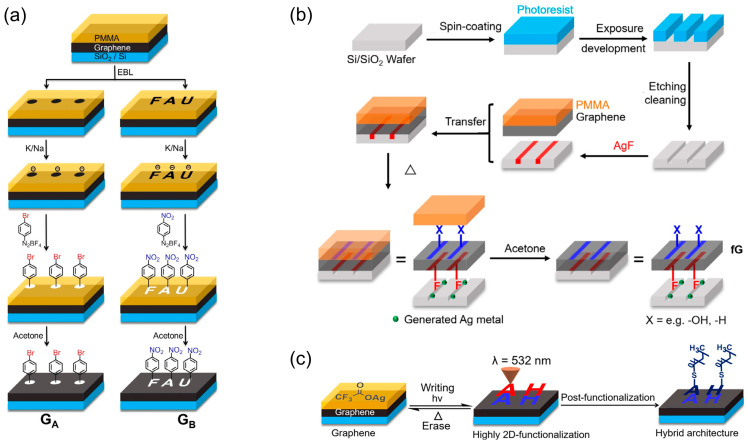
Procedures for molecular patterning on graphene. (**a**) General strategy for selective functionalization on SLG. The dark spots and “FAU” insignia indicate areas of uncovered graphene, whereas other sections are protected by orange PMMA. PMMA is removable with acetone, revealing functional groups attached (highlighted in white) to form GA and GB structures. Reproduced with permission [62]. Copyright 2020, Wiley-VCH. (**b**) Realization of the bottom-side patterning of SLG using site-selective fluorination. Reproduced with permission [63]. Copyright 2020, Wiley-VCH. (**c**) Direct laser writing enabling site-specific, photolytic attachment of functional moieties and silver nanoparticle patterning. Reproduced with permission [64]. Copyright 2020, ACS.

In the latest few years, Hirsch and Wei et al. have developed a series of approaches for covalent patterning on single-layer graphene (SLG) [57,62,63,64,65,66]. The general strategy includes adopting electron beam lithography (EBL) to etch patterns on a poly(methyl methacrylate) (PMMA) resist-coated graphene layer (see Figure 4a), facilitating targeted functionalization of exposed graphene domains [62]. Typically, graphene on a SiO_2_/Si substrate is masked with PMMA; subsequent EBL patterning generates windows through which select graphene regions can be covalently modified by specific reagents. PMMA can be removed by acetone afterwards. A widely explored reaction is the spatially resolved attachment of aryl groups on graphene through an EBL-guided radical reaction with aryl diazonium compounds, where only the graphene areas unprotected by the PMMA resist undergo reaction. A successful protocol for efficient functionalization has been developed, including graphene activation via alkali metals (e.g., a Na/K alloy), which significantly enhances its electrophilic reactivity [65]. In addition, the technique extends to the functionalization of graphene’s underside (see Figure 4b): a patterned substrate embedded with AgF acts as a fluorination agent, allowing for selective bottom-side modifications [63]. Furthermore, emerging research demonstrates the substrate’s pronounced influence on graphene functionalization efficiency within such patterning systems. Graphene exhibited suppressed functionalization on hexagonal boron nitride (hBN) compared to silicon SiO_2_ [67]. This substrate effect provides a strategy for spatially tuning the functionalization degree of graphene.

Laser-induced patterning has also proven efficacious. Direct laser writing offers a straightforward route to 2D functionalization, and silver trifluoroacetate was employed for reaching an unparalleled level of functionalization on graphene (see Figure 4c) [64]. Laser-mediated photolysis of silver trifluoroacetate enables in situ generation of trifluoromethyl radicals, which selectively react with graphene in illuminated zones. This process facilitates precise direct laser writing of spatially resolved patterns onto the graphene surface. Not only does this confer a high degree of functionality to the graphene, but it is also entirely reversible. Post-treatment annealing of these samples facilitates a defunctionalization process, enabling reversible chemical patterning suitable for dynamic chemical information storage systems.

For the diazonium chemistry, another straightforward strategy has been reported for the ambient condition reaction, using diazonium-based grafting ink consisting of an aryl diazonium salt and the dimethyl sulfoxide (DMSO) solvent [68]. The enhanced functionalization stems from DMSO’s dual role: solvating diazonium cations and n-doping graphene, facilitating electron transfer to diazonium cations and leading to the formation of reactive aryl radicals that covalently bind to graphene. Employing a traditional Chinese brush infused with the ink enabled precise writing on graphene films as large as 1 cm^2^, with Raman mapping confirming the pattern. Reaction time precisely dictates the functionalization density, enabling monolayer grafting of the moiety. Remarkably, the ink’s reactivity persists for 14 days at ambient temperature, attributed to the DMSO-diazonium complex’s stability. This reversible functionalization process allows pristine graphene restoration via annealing at 400 °C, permitting subsequent re-application of the ink. This transformative ink simplifies chemical patterning on graphene, paralleling the ease of paper writing.

**Figure 5:**
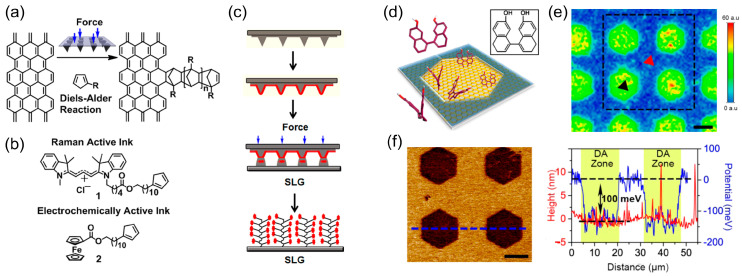
DA reaction induced chemical patterning on graphene. (**a**–**c**) Force accelerated reaction with CP. Reproduced with permission [69]. Copyright 2013, ACS. (**d**–**f**) Reaction by soaking graphene in solution of *cis*-diene. Reproduced with permission [72]. Copyright 2016, ACS. (**a**) Reaction scheme. (**b**) Pattern confirmation: Cy3-tagged Raman-active and ferrocene-tagged electrochemically active ink molecules (1 and 2, respectively) verify the accelerated patterning facilitated by applied force. (**c**) Ink-coated elastomer tip array: a soft, elastic array of tips is coated with a mixture of CP and polyethylene glycol (PEG) (labelled as red color). Upon pressing the inked array against the SLG, the CP adheres while subsequent rinsing removes PEG and excess CP. (**d**) Diagram detailing patterned functionalization of graphene using the hydroxyl-substituted diene (molecular structure displayed in inset) in isopropanol at ambient conditions. (**e**) Raman D peak mapping of graphene after functionalization. (**f**) Surface-scanning kelvin probe microscopy image showing graphene sample’s surface potential, with height (red) and potential (blue) profiles across the dashed line. Scale bars represent 10 μm.

In another interesting attempt (see Figure 5a), cyclopentadienes (CPs) tagged with Raman and electrochemically active moieties (Figure 5b) were covalently grafted onto graphene surfaces using a force-driven Diels−Alder (DA) reaction. This reaction was facilitated by an elastomeric tip array affixed to piezoelectric actuators within an atomic force microscope setup (Figure 5c) [69]. Cycloaddition reactions, characterized by their negative activation volumes, experience pronounced acceleration under applied pressure. These tip arrays, integral to polymer pen lithography [38,70,71], produce precise patterns through the transfer of ink from tips to substrate via an aqueous meniscus. The expansive patterning area (>1 cm^2^) and controlled piezo-actuator movement enable high-throughput and intricate pattern customization. Using Raman-active cyanine and electrochemically active ferrocene CPs, the covalent linkage between the SLG and CPs was investigated via Raman microscopy, cyclic voltammetry, and electronic structure calculations, confirming the presence of micrometer-scale, covalently patterned motifs across large areas. Notably, these reactions proceed under ambient conditions, exploiting a canonical organic transformation, and preserve graphene’s electrical properties, making this method applicable for advanced sensing, electronic, and optical devices.

Parallel to this work, Wan et al. explored DA reaction-mediated graphene functionalization using *cis*-dienes with dihydronaphthalene backbones (see Figure 5d) [72]. By inducing a *cis* conformation featuring a diene moiety in a nonplanar molecular structure, a swift DA reaction time of approximately 5 min was achieved between graphene and the diene functional groups. Sub-micrometer pattern resolution was attained by immersing PMMA-masked graphene in a hydroxyl-substituted *cis*-diene solution at ambient temperature. By increasing the reaction temperature, tunable degrees of functionalization were possible. This DA patterning resulted in p-type doping and improved the electrical conductivity of the graphene films, suggesting potential applications in transparent electrodes. The DA reactivity of *cis*-dienes was retained despite the potential to introduce alternative functional groups on the opposite benzene rings, offering a strategic approach for customized graphene derivatives suitable for sensor technologies. This study provides critical insights into the molecular conformation’s impact on the graphene functionalization process, establishing an efficient and straightforward technique for graphene modification.

For graphene, the surface functionalization also enables the local tuning of the electronic band. Graphene is characterized by a unique electronic structure where the conduction and valence bands converge at distinct points referred to as Dirac cones [56]. The characteristic linear dispersion of electrons near these points imparts graphene with exceptional carrier mobility, laying the foundation for high-performance electronic devices [55]. However, the inherent lack of a band gap complicates the direct utilization in switchable electronic components such as transistors, wherein off states are essential. Nonetheless, the bulk functionalization can undermine spatial selectivity and compromise graphene’s intrinsic electronic properties, causing detrimental impacts. The uncontrolled bulk functionalization can significantly impair charge carrier mobility and disrupt the symmetry of electron-hole transport, thereby negating some of graphene’s most prized attributes. This underscores the necessity for innovative strategies that enable precise functionalization without negating the material’s high carrier mobility [73]. On the contrary, well-designed nanopatterning can forge quasi-one-dimensional channels that exhibit semiconducting behavior while concurrently preserving high electron mobility within these channels. The implications of such structurally defined functionalization present tangible avenues for the creation of microelectrode architectures suited for catalytic and sensing capabilities in electronic and optoelectronic device fabrication. Our reviewed works showcase the precise manipulation of graphene’s surface chemistry can engender ordered or custom patterns of functional sites capable of inducing a band gap while maintaining 1D electronic pathways, allowing for semiconducting properties and good charge mobilities.

Besides SLG, twisted bilayer graphene (TBG) can be an intriguing substrate for patterning. Recent advancements demonstrate the atomic-scale customization of TBG via top-down and bottom-up EBL, conducted at temperatures between 1050 and 1150 °C and confirmed with an aberration-corrected scanning transmission electron microscope [74]. This process strategically employs a focused electron beam to displace carbon atoms from the TBG lattice, creating anchoring sites for heteroatoms like copper. The controlled thermal environment facilitates the mobilization of these foreign atoms from proximal sources across the TBG substrate. Correspondingly, the electron beam induces the selective substitution of graphene carbon with migrating adatoms. The interplay between substrate temperature, adatom mobility, and vacancy dynamics was elucidated through ab initio simulations, underscoring the nuanced control over material transport and integration. This methodology heralds a leap forward in atomic-scale engineering with e-beams, paving the way for precise dopant patterning that can harmonize with inherent features of TBG for advanced material functionalities. It is expected that more chemical functionalization works will be performed on TBG or other graphene derivatives.

## 4. Chemical Patterning on Polycyclic Aromatic Rings

**Figure 6:**
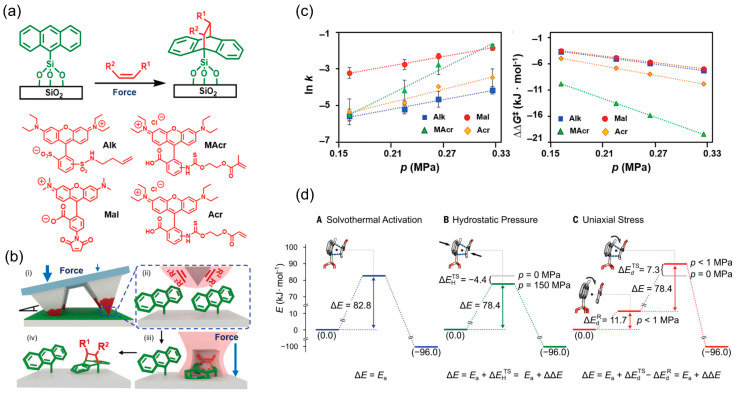
Force-induced DA reactions on anthracene monolayer. (**a**) Schematic of DA cycloaddition between anthracene and dienophiles under mechanical force. Illustrated structures of dienophiles Alk, Mal, MAcr, and Acr. (**b**) The process of elastomeric tip arrays applying a dienophile and PEG ink (red) to an anthracene-coated surface under forces): (i) ink transfer at varying angles; (ii) creating areas of differential force—marked by thick arrows for high force and thin arrows for low force; (iii) these regions act as nanoreactors and increase the speed of DA cycloaddition reactions under applied force; (iv) post-rinse, only the molecules covalently bonded remain. (**c**) Reaction activation parameters on anthracene monolayers, featuring ln(reaction rate) and the change in free energy of activation vs. force for four dienophiles. (**d**) Comparative potential energy profiles under pure solvothermal conditions, hydrostatic pressure, and uniaxial mechanochemical stress. Reproduced with permission [75]. Copyright 2023, AAAS.

Anthracene refers to a polycyclic aromatic hydrocarbon with three benzene rings. Very recently, Braunschweig et al. explored the kinetics and thermodynamics of force-accelerated [4 + 2] DA cycloadditions involving anthracene (Figure 6a) [75,76]. The study employed monolayer configurations to bypass the complexities inherent in bulk mechanochemical processes, such as those encountered in ball milling. Employing elastomeric pyramidal tip arrays (Figure 6b), fluorescently tagged dienophiles were mechanically driven into anthracene monolayers covalently attached to silica wafers. The mechanochemical rate enhancements, notably over 10-fold at low pressures, offered insights into the impact of dienophile electronic and steric variations on the reaction kinetics, distinguished from solvothermal reactivity trends.

In this patterning reaction, applied pressure reduces the reaction energy of the system, albeit to differing degrees. The dienophiles (molecular structures presented in Figure 6a), varying in electron demand and steric bulk—specifically an alkene (Alk), maleimide (Mal), methacrylate (MAcr), and acrylate (Acr)—revealed that minimal force facilitated maximal Mal adduct formation (see red dots in Figure 6c), aligning with electron-demand convention in DA reactions—the presence of electron-withdrawing groups on the dienophile reduces the activation energy. Contrastingly, MAcr exemplified the most significant kinetic change (see yellow diamonds in Figure 6c). Activation energies followed the sequence MAcr > Acr > Alk > Mal, indicative of greater mechanochemical sensitivity for reactions with higher intrinsic barriers. Mechanically applied uniaxial pressure, unlike isotropic hydrostatic conditions, selectively distorts reactants and transition states, seemingly modulating the potential energy surface unique to mechanochemical contexts (see Figure 6d). This implies a broader scope for mechanochemical phenomena than previously considered, irrespective of molecular intricacies. This groundbreaking investigation not only positions surface mechanochemistry as a robust probe for reaction dynamics but also advocates for mechanochemical synthesis as an avenue for green chemical manufacturing. It is expected that multiple functional groups can be feasibly “stamped” on to specific position of the nanocarbon-based surface for various applications in sensing, nanoelectronics and other purposes.

## 5. Outlook and Perspective

Besides the extraordinary effect on the substrate generating novel electronic and quantum properties as illustrated in Section 2 and Section 3, the introduction of various molecular species in the designed surface can facilitate the emergence of spatially defined functionalities, which may result in hybrid structures unseen in normal materials. The induced moieties can be luminescent, electrochemically active, catalytic, bio-active, Raman-active, or with alternated hydrophilic–hydrophobic or electron-donating–electron-withdrawing properties. These advancements open new horizons for nanocarbon-based materials and devices, and enable advances in chemical sensing, spectral fingerprints, and nanoelectronics.

**Figure 7:**
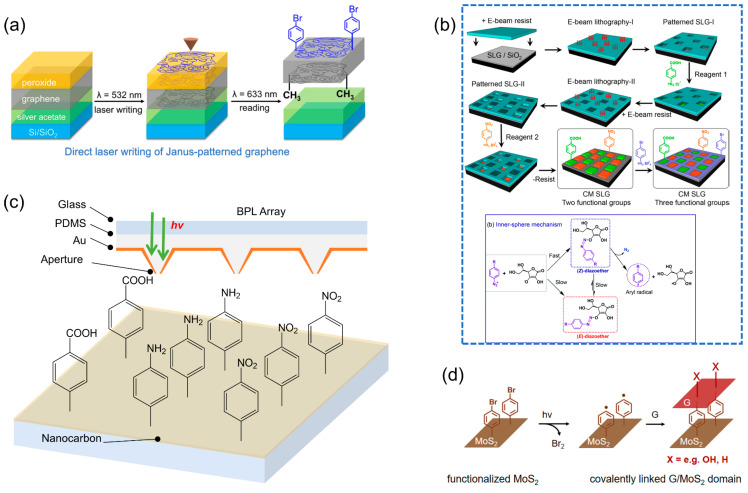
Multiple chemical component patterning on nanocarbon. (**a**) A one-step laser writing method for covalent Janus patterning on graphene. This technique uses simultaneous laser-induced photolysis of dual photosensitizers on opposite sides of the graphene, producing localized radicals and selective addend attachment. Reproduced with permission [77]. Copyright 2022, ACS. (**b**) Diagram of the stepwise chemical patterning of SLG via self-limiting diazoether reactions, enabling the grafting of three distinct types of molecules. The inner sphere mechanism of the diazoether associated with isomer transformation is presented. Reproduced with permission [78]. Copyright 2021, ACS. (**c**) Vison of harnessing substrate-responsive light-driven reactions facilitates precision patterning using near-field techniques such as beam pen lithography for high-resolution and diverse molecular arrangements. (**d**) Depiction of SLG/MoS_2_ covalent heterostructure formation through a laser-induced coupling reaction at the interface. Reproduced with permission [83]. Copyright 2022, ACS.

Despite these advances, more endeavors are required to address current challenges and explore new frontiers. We’d like to share more exciting visons at the forefront of this field. For most cases, the chemical patterning process is limited to the attachment of a single type of functional group, therefore, an important research direction is to pattern multiple chemical information on one single substrate in an efficient and facial manner. Besides the sequential protocols introduced in Section 3, a one-step approach was recently reported for the simultaneous bifunctional patterning of graphene via a synergistic photolithographic and laser-assisted methodology (Figure 7a), enabling the covalent attachment of functional moieties on the opposite sides of the basal plane, resulting in Janus-type graphene [77]. This was accomplished by employing distinct photosensitizers—silver acetate and parabromo-benzoyl peroxide (BBPO)—on opposite side of the graphene sheet. Upon targeted laser irradiation, these agents induce local photolytic reactions, generating highly reactive CH_3_—and BrC_6_H_5_—radicals that selectively bind to the graphene in the illuminated zones, thereby granting precise control over the pattern geometry. This work allows for an accurate and arbitrary manipulation of the pattern shape as demanded on both sides of graphene within only one step.

In another attempt, a combined top-down and bottom-up approach was used to spatially control the patterning of diverse functional groups on one side of SLG, as illustrated in Figure 7b [78]. Initial patterning involved EBL to form square motifs, followed by the first covalent attachment step. Subsequently, the surface was coated with EBL resist and subjected to a secondary lithographic step, ensuring non-overlap with the initial pattern, thereby protecting previously functionalized regions. A second, distinct diazonium salt enabled further covalent modification after resist removal. Lastly, a third diazonium salt was introduced for the final functionalization step, employing functional groups with varying electron-withdrawing capabilities: nitrophenyl (strong), carboxyphenyl (moderate), and bromophenyl (weak). The core reaction chemistry employed in this process is the diazoether chemistry, but in a different way compared with the one discussion in Section 2. As shown in Figure 7b, in an inner-sphere process, aryldiazonium salt reactions with ascorbate ions occur in a tri-step process [79]: (i) fast *Z*-diazoether synthesis; (ii) *Z*-diazoether’s partial decomposition yielding reduction products; (iii) slow *Z*-to-*E* isomerization, with the *E*-isomer being thermodynamically favored. Therefore, the in situ-formed *Z*-isomer can be immediately used as aryl radical precursors for covalently modifying nanocarbon surfaces. In addition, leveraging the self-limiting nature of this grafting chemistry, pattern thickness was precisely maintained at approximately 1 nm, affording an extra dimension of structural control orthogonal to the substrate surface. A carefully selected array of surface analysis techniques was utilized for in-depth examination of the ultrathin covalent patterns, unequivocally verifying the formation of distinctly segregated functional motifs on the graphene basal plane. The simplicity of this approach renders it easily adaptable to a broad spectrum of nanocarbon materials.

Advanced molecular patterning techniques hold great promise for high-resolution, multiple-component decoration of nanocarbon materials. The tunable nature of OCCs associated with s-SWCNTs makes them ideal for controlled and selective functionalization, enabling the attachment of various molecular groups to the host surface. Exploiting light-driven reactions responsive to the substrate instead of the molecule opens avenues for utilizing near-field techniques like beam pen lithography (Figure 7c) for simultaneous precision patterning [80,81,82]. Integration with a microfluidic system could facilitate the simultaneous delivery of distinct molecular precursors on a single substrate, paving the way for the creation of high-resolution, spatially defined multiple component patterns in a facial way. This method holds promise for generating multi-functional molecular platforms with custom-tailored patterns, advancing applications such as multiplexed sensing, and providing the means for the precise modulation of any location on an s-SWCNT substrate to create the desired quantum well structures.

In addition to the rich class of the chemical species available for patterning, the nanocarbon substrate can be potentially extended to other conjugated or partially conjugated counterparts, such as glassy carbon [84], highly oriented pyrolytic graphite [85,86], fullerene, carbon fiber and other types of carbon-based films. Furthermore, the pattern capability will enable the construction of nanocarbon-based heterostructures. Utilizing laser-writing methods alongside a prepatterned MoS_2_ foundational layer, selective synthesis of covalent graphene/MoS_2_ heterostructures was recently achieved [83], replicating the underlying template’s pattern (Figure 7d). This process allows for concurrent covalent bonding at the interface and the transference of topographic designs, streamlining the production of heterostructures at scale. This concept of covalent assembly shows promises for expanding into a wide range of bilayer or multilayer covalent heterostructures, which is crucial for the future of material design and engineering innovations. Therefore, the knowledge and advancement of the pattern on nanocarbon can be employed to create more complex and advanced heterostructures.

## 6. Summary

This review critically assesses the recent advancements in chemical patterning on nanocarbon platforms, with a focus on CNTs, graphene, and related conjugated aromatic systems. Notably, significant progress has been made within the past few years as interdisciplinary integration of nanotechnology and surface chemistry has propelled the evolution of low-dimensional carbon materials with completely tunable physical and chemical properties. The fundamental principles for spatially resolved functionalization of nanocarbons have been well established, and multiple types of chemistries have been recalled to life from traditional organic textbooks and vividly performed on nanocarbon surfaces. The effect of the surface functionalization on the substrate material is enormous. Driven by a deep comprehension of fundamental physics, chemistry, and materials science, scientists have been able to successfully interpret the landmark effect of the functionalization, such as opening up the bandgap [7,87], or creating local quantum wells [44,45]. This is attributed to the unique quantum structure of the low-dimensional nanocarbons.

Despite ongoing and future challenges, the field of spatial selective functionalized nanocarbons is primed to enable breakthrough science and novel applications across diverse fields. The potential of these well-designed materials in electronics, catalysis, sensing, photonics, nanoscience, and quantum technology is significant and poised for exploration and exploitation. This field possess tremendous potential and is ready to embrace more exciting breakthroughs.

## Data Availability

Not applicable.
